# The Adipokine Axis in Heart Failure: Linking Obesity, Sarcopenia and Cardiac Dysfunction in HFpEF

**DOI:** 10.3390/ijms27020612

**Published:** 2026-01-07

**Authors:** Luka Komić, Jelena Komić, Nikola Pavlović, Marko Kumrić, Josipa Bukić, Iris Jerončić Tomić, Joško Božić

**Affiliations:** 1 Department of Family Medicine, Split-Dalmatia County Health Center, 21000 Split, Croatia; luka.komic@mefst.hr (L.K.); jelena.kelam@mefst.hr (J.K.); 2 Department of Pathophysiology, University of Split School of Medicine, 21000 Split, Croatia; 3 Laboratory for Cardiometabolic Research, University of Split School of Medicine, 21000 Split, Croatia; 4 Department of Cardiovascular Diseases, University Hospital of Split, 21000 Split, Croatia; 5 Department of Pharmacy, University of Split School of Medicine, 21000 Split, Croatia; 6 Department of Laboratory Medicine and Pharmacy, Faculty of Medicine, Josip Juraj Strossmayer University of Osijek, 31000 Osijek, Croatia; 7 Department of Public Health, University of Split School of Medicine, 21000 Split, Croatia; iris.jeroncic.tomic@mefst.hr

**Keywords:** adipokine axis, heart failure, obesity-related inflammation, sarcopenia and muscle metabolism, cardiometabolic remodeling

## Abstract

Obesity, sarcopenia, and heart failure with preserved ejection fraction (HFpEF) constitute an interconnected clinical triad driven by multisystem mechanisms centered on the adipokine axis. Adipose tissue, now recognized as a dynamic endocrine organ, undergoes pathological remodeling in obesity, characterized by hypoxia, chronic low-grade inflammation, and dysregulated adipokine secretion. These changes impair endothelial function, promote myocardial fibrosis, and disrupt skeletal muscle metabolism, thereby linking cardiometabolic and musculoskeletal dysfunction. This review integrates current evidence on homeostatic adipokines, such as adiponectin, apelin, and omentin, that preserve vascular and muscular resilience, as well as stress-inducible adipokines, such as leptin, resistin, and GDF15, that reflect or amplify metabolic and inflammatory injury. A maladaptive adipokine milieu associates with a self-reinforcing cycle of endothelial dysfunction, myocardial stiffening, and muscle atrophy that characterizes s HFpEF in the context of obesity and sarcopenia. We further discuss emerging translational applications, including diagnostic and prognostic adipokine signatures, targeted modulation of adipokine pathways, and the therapeutic impact of GLP-1 receptor agonists on adipose–cardiovascular–muscle crosstalk. Remaining challenges, including the adiponectin paradox and pleiotropic adipokine effects, highlight the need for precision-medicine approaches integrating multimodal biomarker profiling with cardiometabolic and musculoskeletal phenotyping.

## 1. Introduction

Obesity is a major global health challenge and a central driver of cardiometabolic disease. Beyond its mechanical and hemodynamic effects, obesity is characterized by chronic low-grade inflammation and endocrine dysregulation, mainly mediated by adipokines, bioactive molecules secreted by adipose tissue that exert both local and systemic actions [1,2,3,4,5,6]. In this context, adipose tissue is now recognized as a dynamic endocrine organ that influences metabolic homeostasis, vascular tone, and myocardial structure through a complex secretory profile that extends far beyond energy storage [4,5,6,7].

The recognition of adipose tissue as an endocrine system began with the identification of the *ob* gene in 1994, which encodes leptin, a hormone secreted by adipocytes that regulates appetite and energy balance through hypothalamic signaling pathways [8]. Soon thereafter, adiponectin was identified as another abundant adipocyte-derived protein with insulin-sensitizing, anti-inflammatory, and vasculoprotective properties [9,10]. The subsequent cloning of adiponectin receptors (AdipoR1 and AdipoR2) in 2003 revealed receptor-mediated pathways involving AMP-activated protein kinase (AMPK) and peroxisome proliferator-activated receptor-α (PPAR-α) that link adipokine signaling to lipid oxidation, mitochondrial biogenesis, and nitric oxide (NO) bioavailability [11,12]. Collectively, these discoveries established adipokines as endocrine mediators of systemic energy homeostasis. At the same time, later research delineated their influence on vascular biology and myocardial remodeling, thereby positioning them as potential contributors to cardiovascular disease [4,5,6,7,11,12].

As evidence accumulated, it became clear that adipose-derived signaling extends beyond the myocardium and vasculature to affect skeletal muscle metabolism, influencing insulin sensitivity, substrate utilization, and inflammatory tone [2,13]. This multiorgan communication, linking adipose tissue, skeletal muscle, and the heart, provides the biological basis for what is now conceptualized as the adipokine axis: a regulatory network integrating metabolic, inflammatory, and structural responses across organ systems. Dysregulation within this axis appears associated with developmentof HFpEF and the parallel emergence of sarcopenia in metabolic disease [2,3,4,5,6,13].

In this review, we adopt an integrative framework in which obesity and ectopic fat depots (notably visceral and epicardial adipose tissue) promote a shift in the adipose secretome toward pro-inflammatory, pro-oxidative, and pro-fibrotic signaling. These signals act on the coronary microvascular endothelium, fostering endothelial activation and impaired NO bioavailability; on cardiomyocytes, increasing passive stiffness and energetic inefficiency; on cardiac fibroblasts, amplifying extracellular matrix deposition; and on skeletal muscle, aggravating insulin resistance and sarcopenic trajectories that further limit exercise capacity [4,5,6]. Within this framework, adipokines are conceptualized as shared molecular mediators linking cardiometabolic dysfunction to the HFpEF phenotype, while acknowledging that individual adipokines exhibit context-dependent and tissue-specific effects and may represent compensatory responses rather than unidirectional drivers of disease. Regarding the type of heart failure, HFpEF was selected as the primary focus because it represents a syndrome in which obesity and metabolic co-morbidities are disproportionately prevalent, and where a prominent mechanistic paradigm implicates coronary microvascular endothelial activation, systemic inflammation, and myocardial stiffening [3,4]. Beyond observational associations, accumulating genetic and phenotyping evidence supports a reciprocal relationship between adiposity and HFpEF. Mendelian randomization studies suggest that central obesity and adverse fat distribution exert causal effects on cardiac structure and heart failure risk [14]. Detailed phenotyping of HFpEF cohorts consistently demonstrates a high burden of sarcopenia and sarcopenic obesity, marked by excess visceral adiposity in conjunction with reduced skeletal muscle mass and impaired muscle function. These abnormalities are closely linked to exercise intolerance, metabolic dysregulation, and diastolic dysfunction, rather than to systolic impairment [15,16]. While several adipokines have been studied across the full heart failure spectrum, their interpretation may differ between HFpEF and HFrEF due to differences in ventricular remodeling, systemic congestion, and neurohormonal activation. Furthermore, the predominant etiological context differs between the two phenotypes: HFpEF most frequently arises in the setting of obesity, metabolic dysfunction, hypertension, aging, and systemic inflammatory–endothelial dysregulation, whereas HFrEF is more commonly linked to ischemic injury, cardiomyopathies, or primary myocardial loss [5]. Because these distinct upstream drivers likely modulate adipokine biology in fundamentally different ways, we deliberately focus this review on HFpEF rather than HFrEF, where the metabolic–inflammatory context renders adipokine signaling particularly relevant to pathophysiology and clinical interpretation.

The review is based on a structured literature search in PubMed/MEDLINE and Scopus. The initial search was limited to English-language articles published over the past 10 years. It combined MeSH terms and free-text keywords related to adipokines, obesity, sarcopenia, and HFpEF, including “Obesity” [Mesh], “Heart Failure, Diastolic” [Mesh], “Sarcopenia” [Mesh], and “Adipokines” [Mesh]. After searching different combinations of these terms, relevant publications were identified, with priority given to meta-analyses, systematic reviews, and randomized clinical trials. Additional studies were retrieved by screening the reference lists of key articles.

This review examines the adipokine axis as a mechanistic and translational link between obesity, sarcopenia, and cardiac dysfunction, emphasizing integrated pathophysiology, biomarker potential, and therapeutic implications.

## 2. The Adipokine Axis as a Multisystem Regulatory Network

Adipose-tissue expansion in obesity associates with a qualitative transformation of this organ’s biology, shifting it from a passive energy reservoir into a metabolically active endocrine and immune interface that communicates stress to distant tissues. In visceral and epicardial depots, adipocyte hypertrophy increases diffusion distance and oxygen demand, creating relative hypoxia that stabilizes hypoxia-inducible factor-1α (HIF-1α) and induces expression of pro-inflammatory and profibrotic genes [5,6,17,18,19,20]. In murine and human adipose tissue, HIF-1α activation up-regulates monocyte chemoattractant protein-1 (MCP-1/CCL2) and CCL5, which orchestrate monocyte recruitment and macrophage retention within expanding adipose depots [5,6,18,19,20,21]. Although adipocytes do not execute fibrosis directly, these secreted mediators orchestrate macrophage recruitment, fibroblast activation, and extracellular matrix deposition, thereby promoting adipose tissue fibrosis. In epicardial adipose tissue (EAT), similar hypoxic signaling increases secretion of these chemokines directly toward the adjacent myocardium, establishing a paracrine inflammatory gradient that fuels microvascular dysfunction and myocardial fibrosis [5,6,20,22,23,24].

These observations are substantiated by direct experimental evidence. Lee et al. demonstrated that CCL2 (also known as MCP-1) and *CCL5* gene expression rise markedly in mesenteric adipose tissue of obese *KKAy* mice [21]. Furthermore, Dahlman et al. confirmed in human subjects that MCP-1/CCL2 is uniquely overexpressed among chemokines in adipose tissue of obese individuals [18]. Sartipy and Loskutoff reported similar over-expression of MCP-1/CCL2 mRNA in adipose tissue of genetically obese mice [19]. Complementary human data show that EAT thickness correlates with MCP-1/CCL2 concentrations and left-ventricular remodeling [20]. Collectively, these data establish that adipocyte hypertrophy and inadequate angiogenesis trigger HIF-1α–dependent chemokine signaling, leading to macrophage infiltration and chronic low-grade inflammation, the initiating event in adipokine-axis dysregulation. Critically, while rodent models excel in causality, human data are cross-sectional, limiting temporal inference—yet consistency across species strengthens the hypoxia-inflammation link as adipokine axis initiator [18,19,20,21].

Within these inflamed depots, macrophages polarize toward an M1 phenotype, releasing tumor-necrosis factor-α (TNF-α), interleukin-6 (IL-6), resistin, and retinol-binding protein-4 (RBP4), while adipocytes down-regulate adiponectin and omentin expression [4,5,6,7,17,18,19,20,21,22,23,24]. Foundational experimental studies, in both humans and mice, demonstrated that adipose expression of TNF-α drives obesity-associated insulin resistance; resistin and RBP4 link adipocyte inflammation to systemic metabolic and vascular injury; and reduced adiponectin and omentin expression attenuates AMP-activated protein kinase and endothelial nitric oxide synthase (eNOS) signaling [25,26,27,28,29,30,31]. This remodeling of the adipokine secretome establishes an endocrine environment that blunts NO bioavailability and promotes oxidative stress [32,33,34]. TNF-α and resistin suppress eNOS phosphorylation and stimulate reactive oxygen species (ROS) generation via NADPH oxidase activation. At the same time, xanthine oxidoreductase-derived superoxide further consumes NO, compounding endothelial dysfunction [32,33,34]. The resulting reduction in NO-stimulated soluble-guanylyl-cyclase activity decreases cGMP-protein-kinase G (PKG) signaling in cardiomyocytes, leading to titin hypophosphorylation, increased sarcomeric stiffness, and impaired myocardial relaxation—the cellular substrate of diastolic dysfunction in HFpEF [3,23,35].

EAT epitomizes the paracrine potential of this process. Sharing microvasculature with the underlying myocardium and lacking fascial separation, EAT provides direct humoral access for adipokines to coronary microvessels [22]. In obesity and diabetes, its inflammatory transformation results in increased secretion of leptin, resistin, and MCP-1/CCL2, along with diminished adiponectin secretion [3,4,5,22,23,24]. This secretory imbalance activates coronary endothelial ICAM-1 and VCAM-1, recruits macrophages and T cells, and stimulates fibroblast TGF-β/SMAD signaling, culminating in perivascular and interstitial fibrosis in mice [3,23,36].

Parallel alterations develop in skeletal muscle, the second major effector within the adipokine axis. Chronic exposure to the inflammatory adipokine milieu suppresses AMPK–SIRT1–PGC-1α signaling, decreases mitochondrial biogenesis and β-oxidation, and induces FoxO3-driven expression of atrogin-1 and MuRF1, accelerating proteasomal degradation of contractile proteins [13,37,38,39]. Mitochondrial dysfunction and anabolic resistance lead to sarcopenic remodeling with reduced oxidative capacity and capillary density. These muscular changes feed back on the adipokine network by diminishing energy expenditure and substrate clearance, thereby sustaining hyperleptinemia and hyperresistinemia [13,39].

**Figure 1:**
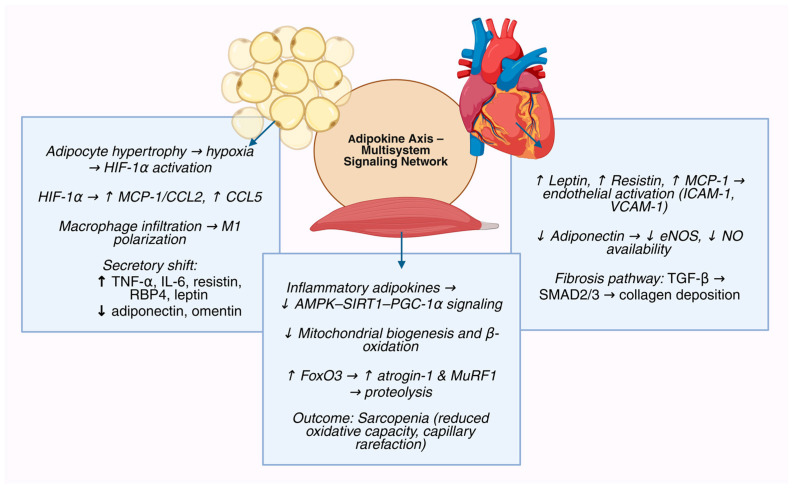
The adipokine axis is a multisystem regulatory network connecting adipose tissue, the myocardium, and skeletal muscle. Adipocyte hypertrophy in visceral and epicardial depots induces local hypoxia, stabilizes HIF-1α, and drives transcription of MCP-1/CCL2 and CCL5, which promote monocyte recruitment and M1 macrophage polarization. Inflamed adipose tissue shifts its secretory profile toward increased TNF-α, IL-6, resistin, and RBP4, and toward decreased adiponectin and omentin, establishing a pro-inflammatory and vasculotoxic endocrine milieu. In EAT, this imbalance exerts direct paracrine effects on the adjacent myocardium, activating coronary endothelial ICAM-1 and VCAM-1, stimulating TGF-β/SMAD-mediated fibrosis, and reducing NO bioavailability. Impaired NO–sGC–cGMP–PKG signaling decreases titin phosphorylation, increasing cardiomyocyte stiffness and contributing to diastolic dysfunction characteristic of HFpEF. Parallel inflammatory signaling in skeletal muscle suppresses AMPK–SIRT1–PGC-1α pathways, impairs mitochondrial biogenesis, increases FoxO3-driven ubiquitin ligases atrogin-1 and MuRF1, and accelerates proteolysis, culminating in sarcopenia. Muscle metabolic failure further reinforces systemic adipokine dysregulation, sustaining hyperleptinemia and hyperresistinemia. Together, these inter-organ interactions define the adipokine axis as a self-reinforcing network linking obesity, myocardial stiffening, and muscle atrophy. Created in BioRender. Kumric, M. (2026). https://BioRender.com/3htw17l (accessed on 15 November 2025).

At the systemic level, these disturbances manifest as a reproducible endocrine signature: elevated leptin, resistin, and RBP4 coupled with reduced adiponectin and omentin [4,5,6,7,25,26,27,28,29,30]. This signature integrates inflammatory, oxidative, and metabolic stress across adipose tissue, skeletal muscle, and myocardium, producing the characteristic triad of obesity, sarcopenia, and HFpEF. Conceptually, it defines the adipokine axis as a multiorgan feedback system that synchronizes typical nutrient status with vascular and muscular function but, when dysregulated, drives a self-reinforcing cycle of endothelial inflammation, myocardial stiffening, and muscular atrophy [3,4,5,6,7,13,17,18,19,20,21,22,23,24]. The key molecular pathways through which adipose-tissue inflammation propagates myocardial and skeletal-muscle dysfunction are summarized in Figure 1.

## 3. The Adipokine Axis as the Integrator of Obesity, Sarcopenia, and HFpEF—An Adipokine-Centric Synthesis

The syndromic convergence of obesity, sarcopenia, and HFpEF emerges from a continuous endocrine–paracrine network in which adipokines reshape endothelial tone, cardiomyocyte stiffness, and skeletal-muscle metabolism in lockstep. Rather than isolating organ phenotypes, we trace this triad through prototypical homeostatic adipokines that restrain vascular and myocardial inflammation, stress-inducible adipokines that gauge and compensate for metabolic/cellular stress, and injurious adipokines that propagate fibrosis and metabolic inflexibility [4]. What follows is an adipokine-by-adipokine narrative in which each signal threads through adipose tissue, the coronary microvasculature, myocardium, and skeletal muscle, making the mechanistic unity of the triad explicit.

### 3.1. Homeostatic Adipokines That Preserve Endothelial–Myocardial–Myocellular Resilience

In healthy adipose tissue, adiponectin sustains endothelial NO bioavailability via cAMP–eNOS signaling and restrains NF-κB–mediated inflammation; these effects are demonstrated to maintain coronary microvascular compliance and PKG signaling in cardiomyocytes, consistent with reduced titin-based stiffness and preserved lusitropy [28,32,35]. In skeletal muscle, AdipoR1/R2–AMPK–SIRT1–PGC-1α signaling preserves mitochondrial biogenesis and lipid oxidation, averting the anabolic resistance that seeds sarcopenia [11,12,13,38]. In early obesity, adiponectin falls, particularly in inflamed EAT, and this reduction associates with reduced vasodilator tone and increased pro-oxidant cytokine action on endothelium and myocytes—an early lesion that couples early metabolic dysfunction to diastolic dysfunction and impaired exercise tolerance [7,22,23,32]. Notably, this early adiponectin deficiency stands in contrast to the “adiponectin paradox” described in established HFpEF, in which circulating adiponectin levels increase despite progressive microvascular, metabolic, and diastolic impairment, a phenomenon discussed in greater detail later in Section 3.4.

Apelinergic signaling complements adiponectin by stimulating endothelial NO and activating AMPK/Akt, reducing arterial stiffness and microvascular inflammation, and thereby lowering diastolic load; in skeletal muscle, apelin improves mitochondrial function and angiogenesis, countering sarcopenic fatigue [29,32,40]. In cardiometabolic HFpEF, apelin expression is typically suppressed within epicardial and perivascular adipose tissue, paralleling systemic endothelial dysfunction. Recent proteomic and imaging data from the PROMIS-HFpEF study confirm that increased EAT burden correlates with coronary microvascular dysfunction, diastolic stiffness, and inflammatory signatures, reinforcing the view that adipose–vascular cross-talk contributes to myocardial remodeling [29,32,40,41].

Omentin-1, secreted predominantly from visceral and epicardial adipose tissue, enhances insulin sensitivity and promotes endothelial NO–dependent relaxation via AMPK–eNOS activation [30]. In obesity and inflamed EAT, its expression declines, fostering endothelial dysfunction and metabolic stress [30,42]. In human HFpEF cohorts, circulating omentin-1 levels are significantly reduced and inversely correlate with markers of inflammation and diastolic stiffness, aligning the loss of this homeostatic adipokine with the cardiometabolic HFpEF phenotype [43].

SFRP5 buffers non-canonical Wnt5a signaling, limiting endothelial and macrophage inflammatory activation and reducing post-ischemic myocardial inflammation; in obesity, SFRP5 levels decline while Wnt5a expression rises, shifting the vasculo-myocardial milieu toward adhesion-molecule induction, leukocyte recruitment, and fibroblast activation [44,45,46]. The resulting loss of this anti-inflammatory brake integrates metabolic stress in adipose tissue with microvascular inflammation and myocardial fibrotic remodeling, key hallmarks of the HFpEF phenotype [44,45,46,47,48].

Recent work positions CTRP9 as a vasculo-myocardial integrator that couples adipose signals to endothelial repair and microvascular tone. In endothelial cells and endothelial progenitors, CTRP9 activates AMPK-dependent pathways that converge on HDAC7/p38/MEF2 and eNOS, thereby promoting angiogenesis, migration, and tube formation and rescuing high-glucose–induced dysfunction [31,49,50]. Clinically, circulating CTRP9 concentrations are lower in coronary artery disease by meta-analysis, aligning reduced CTRP9 tone with atherosclerotic burden and endothelial impairment [51]. Beyond endothelium, CTRP9 restrains atherogenesis by enhancing macrophage autophagy (USP22↑/SIRT1 maintenance) and opposing foam-cell formation; conversely, promoter hypermethylation of CTRP9 in vascular smooth-muscle cells favors lipid deposition and ER-stress–driven dysfunction, mechanistically linking cardiometabolic stressors (e.g., homocysteine) to vascular injury [52,53]. In the heart, loss of CTRP9 exacerbates fibrosis and remodeling in diabetic cardiomyopathy through YAP-modulated autophagy, highlighting its therapeutic potential in HFpEF phenotypes marked by microvascular inflammation and fibrosis [54]. Collectively, current evidence supports CTRP9 as a homeostatic adipokine whose decline in metabolic disease removes a critical brake on endothelial inflammation and rarefaction—core processes driving diastolic stiffness.

### 3.2. Stress-Inducible Adipokines That Sense Energetic Strain and Attempt Rescue

FGF21 is induced by nutrient deprivation and mitochondrial stress, functioning as an endocrine signal that enhances fatty-acid oxidation, autophagy, and antioxidant defenses in cardiomyocytes and skeletal muscle. Mechanistically, it activates AMPK–SIRT1–PGC-1α pathways to preserve mitochondrial quality control, attenuate oxidative injury, and maintain metabolic flexibility under stress conditions [55,56,57,58]. Circulating FGF21 concentrations are elevated in patients with diastolic dysfunction and HFpEF, where higher levels correlate with increased filling pressures and impaired exercise capacity, suggesting a compensatory response or an acquired state of FGF21 resistance analogous to that seen in obesity and type 2 diabetes [59,60].

GDF15, a mitochondrial stress-responsive protein, is elevated in response to oxidative stress, endoplasmic reticulum stress, and impaired mitochondrial proteostasis. In experimental systems, cardiomyocytes and skeletal muscle fibers under metabolic strain markedly upregulate GDF15 transcription via ATF4 and CHOP signaling, consistent with its role as a marker of cellular distress and energy imbalance [61,62]. In multimorbid, obese individuals with HFpEF, circulating GDF15 concentrations are consistently elevated and correlate with left-ventricular filling pressures, diastolic stiffness, and impaired exercise tolerance [63,64,65]. Clinical studies, in both HFpEF and HFrEF, demonstrate that elevated GDF15 independently associates with and predicts mortality and rehospitalization, establishing its strong prognostic biomarker value. However, whether GDF15 itself is causally pathogenic or primarily reflects underlying mitochondrial dysfunction and cellular stress remains uncertain. Targeted interventional studies modulating GDF15 in HFpEF are needed to establish causal mechanisms and therapeutic potential [65,66,67].

Beyond the myocardium, GDF15 reflects skeletal-muscle mitochondrial dysfunction and reduced oxidative capacity—hallmarks of sarcopenic remodeling. Experimental and translational evidence show that elevated GDF15 tracks with diminished muscle mass and impaired oxidative phosphorylation [61,68]. Collectively, these findings position GDF15 as a whole-body stress integrator that mechanistically bridges adipose inflammation, muscular bioenergetic failure, and cardiac dysfunction within the obesity–sarcopenia–HFpEF triad [62,66,67].

NAMPT maintains myocardial NAD^+^ and activates SIRT1-dependent cytoprotective programs; cardiac NAMPT overexpression or NMN supplementation reduces ischemia/reperfusion injury and improves stress tolerance, with loss of benefit when SIRT1 is disabled [69,70]. In pressure overload, monocyte-derived extracellular NAMPT (eNAMPT) can transiently bolster myocardial NAD^+^ and preserve SIRT1 activity, supporting hemodynamic compensation [71,72]. Yet chronic elevations of eNAMPT—as a pro-inflammatory cytokine (PBEF/visfatin)—may fuel endothelial activation and fibrosis; hence the dualism of the NAMPT axis: short-term cytoprotection versus long-term inflammatory remodeling if adipose–immune sources dominate [6,73,74].

Irisin, a cleaved product of the fibronectin type III domain-containing protein 5 (FNDC5), exemplifies the molecular dialog between skeletal muscle and adipose tissue. Originally described as a PGC-1α–dependent myokine, it mediates exercise-induced browning of white adipocytes and enhances mitochondrial oxidative capacity through UCP1 up-regulation and AMPK–p38 MAPK activation, thereby increasing systemic energy expenditure and improving insulin sensitivity [75,76,77]. Beyond its metabolic role, irisin exerts pleiotropic cardiovascular actions—augmenting endothelial NO bioavailability, attenuating oxidative stress, and improving endothelial progenitor-cell function [76,77].

In cardiometabolic disease, particularly HFpEF, circulating irisin levels reflect the integrity of muscle–adipose–cardiac cross-talk. Clinical and translational data indicate that lower irisin concentrations accompany obesity, sarcopenia, and systemic inflammation, consistent with impaired myokine signaling and reduced oxidative reserve [78,79]. In HFpEF, irisin deficiency correlates with diastolic dysfunction, increased arterial stiffness, and poor exercise capacity, while experimental administration of recombinant irisin ameliorates endothelial dysfunction and cardiac remodeling by stimulating AMPK–eNOS and Akt–ERK pathways [76,79,80].

Conversely, a subset of reports describes paradoxically elevated irisin levels in advanced or decompensated heart failure, possibly reflecting compensatory up-regulation under oxidative and catabolic stress [78,81]. Lower baseline irisin nevertheless predicts reduced aerobic capacity and adverse outcomes, suggesting that diminished muscle-derived signaling contributes to metabolic inflexibility [79,80]. Within the adipokine–myokine network, irisin complements adiponectin, apelin, and omentin in maintaining endothelial NO bioavailability and mitochondrial efficiency; when this signaling is blunted—as in sarcopenic obesity and HFpEF—the result is compounded endothelial dysfunction, energetic inefficiency, and exercise intolerance, hallmarks of adipokine-axis decompensation [77,79,80].

### 3.3. Injurious Adipokines That Enforce Endothelial Inflammation, Fibrosis, and Anabolic Failure

Hyperleptinemia and leptin spillover from inflamed EAT associate with endothelial and myocardial dysfunction. Within EAT, leptin expression increases in proportion to adipocyte hypertrophy and local macrophage infiltration, serving as a potential paracrine mediator to adjacent myocardium [4,22,23,82,83,84]. In experimental models, leptin is demonstrated to induce ICAM-1 and VCAM-1 expression and promote ROS generation through NADPH-oxidase activation. In humans, chronic hyperleptinemia associates with impaired endothelial NO bioavailability and increased microvascular stiffness, though causality in the multifactorial HFpEF setting remains incompletely established, particularly given acquired leptin resistance via caveolin-1–mediated suppression of receptor signaling in chronic obesity [31].

In parallel, leptin stimulates cardiomyocyte oxidative stress and mitochondrial dysfunction, while engaging PKC–NF-κB pathways that drive apoptosis and inflammatory gene expression—effects demonstrated directly in human EAT–cardiomyocyte co-culture and murine HFpEF models [85].

Resistin, TNF-α, RBP4, FABP4, LCN2. In experimental models, resistin and TNF-α suppress eNOS and activate NADPH oxidase, thereby depleting NO; in humans, elevated resistin and TNF-α associate with impaired endothelial function and microvascular stiffening, though the causal contribution of each mediator remains difficult to establish due to overlapping inflammatory networks [32,34]. RBP4 impairs insulin signaling and augments vascular inflammation, while FABP4 (Fatty Acid Binding Protein 4)—secreted predominantly by adipocytes—correlates with HFpEF remodeling and outcomes, consistent with lipotoxic endothelial–myocardial injury [31,32,86,87,88]. LCN2 (NGAL), an adipose-and neutrophil-derived siderophore-binding glycoprotein, serves as a biomarker of cardiometabolic remodeling. Experimental studies demonstrate that LCN2 is associated with cardiomyocyte hypertrophy and mitochondrial stress, while elevated circulating levels in humans correlate with left-ventricular hypertrophy and diastolic dysfunction; however, whether LCN2 itself is causally pathogenic or reflects systemic inflammatory burden remains to be clarified in chronic kidney disease [89,90]. Meta-analytic data confirm its association with adverse outcomes in acute heart failure, while prospective cohort data support a similar association with major adverse cardiovascular events and heart failure hospitalizations in patients with stable coronary artery disease [91,92]. Collectively, these findings position LCN2 as a biomarker and potential effector linking metabolic inflammation and microvascular dysfunction to the HFpEF phenotype.

Elevated circulating chemerin concentrations predict adverse outcomes and remodeling in chronic heart failure, and correlate independently with arterial stiffness and endothelial dysfunction in cardiometabolic disease [93,94]. Experimental and translational studies show that chemerin signaling promotes oxidative stress, endothelial activation, and lipid-handling disturbances within the myocardium, linking visceral adiposity to diastolic stiffness and microvascular inflammation [95]. Collectively, chemerin functions as a paracrine amplifier of adipose-derived endothelial–myocardial cross-talk central to the HFpEF phenotype.

Autotaxin (ENPP2), secreted by adipocytes and vascular cells, catalyzes the conversion of lysophosphatidylcholine into lysophosphatidic acid (LPA), a bioactive lipid that regulates endothelial permeability, leukocyte adhesion, and fibroblast activation. Experimental inhibition of the ATX–LPA pathway suppresses post-ischemic inflammation and attenuates ventricular fibrosis and remodeling, underscoring its causal role in cardiac injury [96,97]. Clinical studies demonstrate that circulating ATX concentrations are elevated in non-ischemic cardiomyopathy and predict adverse outcomes, highlighting its biomarker potential in fibrotic heart disease [98]. Mechanistic reviews further identify the ATX–LPA–LPP3 axis as a central regulator of myocardial fibrosis, metabolic inflammation, and diastolic dysfunction [99,100]. Given the paracrine proximity of EAT to the myocardium, ATX-derived LPA provides a direct biochemical conduit linking visceral adiposity to microvascular inflammation and fibrotic stiffening characteristic of the HFpEF phenotype.

Matrix-linked adipokines and alarmins (galectin-3, osteopontin, WISP1/CCN4, ST2/IL-33, ANGPT2/ANGPTLs, PAI-1, S100–RAGE). The adipokine axis converges on fibro-inflammatory matrix remodeling.

Galectin-3, secreted primarily by activated macrophages and fibroblasts, promotes collagen cross-linking, fibroblast proliferation, and persistent extracellular-matrix remodeling through TGF-β/Smad-dependent pathways. Experimental inhibition or genetic deletion of galectin-3 prevents adverse remodeling and fibrosis in pressure-overload models, supporting a mechanistic role for galectin-3 in cardiac fibrogenesis [101]. In patients with HFpEF, circulating galectin-3 levels correlate with diastolic stiffness, impaired functional capacity, and adverse outcomes, as demonstrated in the Aldo-DHF trial [102]. Contemporary data consolidate galectin-3 as both a mechanistic driver and a clinically useful biomarker of myocardial fibrosis, with emerging therapeutic potential for targeting macrophage-fibroblast signaling in HFpEF [103].

Osteopontin is a matricellular protein secreted by macrophages and fibroblasts. It links metabolic inflammation to myocardial fibrosis. Acting through integrin and CD44 signaling, it promotes myofibroblast differentiation, collagen deposition, and mitochondrial oxidative stress, leading to diastolic dysfunction [104,105]. Elevated circulating osteopontin levels predict adverse remodeling and outcomes in both HFpEF and HFrEF, underscoring its role as a biomarker and effector of metabolic cardiac stiffening [104,105,106].

WISP1/CCN4 is a stress-responsive matricellular protein that bridges metabolic inflammation and myocardial fibrosis. It activates cardiac fibroblasts and stimulates collagen maturation through integrin/AKT–TGF-β signaling, promoting extracellular-matrix accumulation and ventricular stiffening [107,108,109,110]. sST2 (IL-33 decoy), the soluble interleukin-33 receptor sST2, rises in response to myocardial stretch, inflammation, and fibrosis. By neutralizing IL-33, it attenuates cardioprotective and antifibrotic signaling, thereby promoting ventricular stiffening and adverse remodeling. Elevated sST2 concentrations independently predict hospitalization and cardiovascular death across heart-failure phenotypes, including HFpEF [111,112,113].

ANGPT2 destabilizes endothelial junctions and promotes vascular permeability and inflammation, processes implicated in cardiopulmonary vascular remodeling and HFpEF pathobiology. In parallel, the adipose-inflammation signal ANGPTL2 drives cardiac dysfunction through mitochondrial and energetic derangements and activation of cellular senescence pathways [114,115,116,117,118,119].

PAI-1 is among the few biomarkers repeatedly tied to incident HFpEF rather than HFrEF in cross-cohort analyses, and higher tPA/PAI-1 complexes predict worse outcomes in established HFpEF—linking visceral metabolic aging to microvascular fibrosis [120,121,122,123].

**Figure 2:**
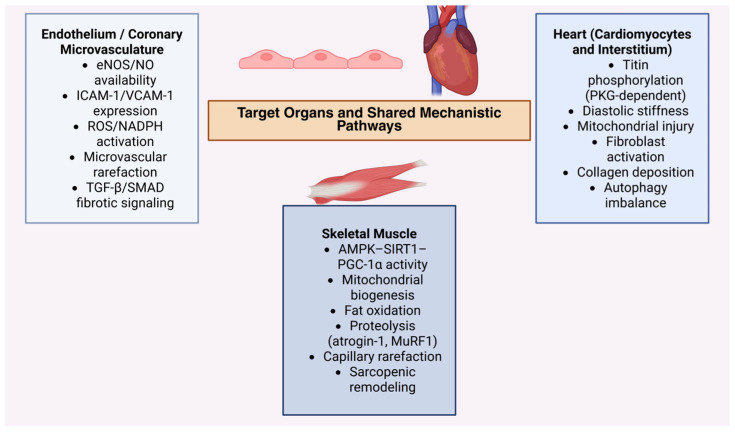
Integrated schematic illustrating how homeostatic, stress-inducible, and injurious adipokines collectively link obesity, sarcopenia, and HFpEF through convergent endothelial, myocardial, and skeletal-muscle pathways. Protective adipokines (adiponectin, apelin, omentin-1, SFRP5, CTRP9) normally sustain NO bioavailability, mitochondrial function, and anti-inflammatory tone. Stress-inducible adipokines (FGF21, GDF15, NAMPT, irisin) sense energetic strain and initiate compensatory metabolic programs. Injurious adipokines (leptin, resistin, TNF-α, RBP4, FABP4, LCN2, chemerin, ATX/LPA, and matrix-linked mediators including galectin-3, osteopontin, WISP1, sST2, ANGPT2, PAI-1, S100/RAGE) propagate ROS production, endothelial activation, fibroblast recruitment, and metabolic inflexibility. Their combined actions converge on impaired NO-sGC-cGMP-PKG signaling, myocardial stiffening, and skeletal-muscle mitochondrial failure—forming the mechanistic foundation of the obesity–sarcopenia–HFpEF phenotype. Created in BioRender. Kumric, M. (2026) https://BioRender.com/my51vze (accessed on 17 November 2025).

Activation of the S100–RAGE axis promotes cardiomyocyte apoptosis, fibroblast activation, and vascular inflammation, and is upregulated in obesity and heart failure—extending the fibro-inflammatory cascade initiated in inflamed adipose tissue [124,125]. A conceptual overview of how homeostatic, stress-inducible, and injurious adipokines integrate endothelial, myocardial, and skeletal-muscle dysfunction within the obesity–sarcopenia–HFpEF triad is illustrated in Figure 2.

### 3.4. Controversies and Evidence Gaps

Despite the expanding literature, research on adipokines in HFpEF remains characterized by substantial heterogeneity and important unresolved knowledge gaps. Across cohorts, reported associations between individual adipokines and clinical outcomes show high variability, reflecting differences in patient populations, comorbidity profiles, adiposity distribution, disease stage, and analytical methodologies. This heterogeneity complicates direct comparison between studies and limits firm causal inference [126,127].

One of the most prominent conceptual challenges is the so-called adiponectin paradox. Clinical counterpoint is that in established HF, including HFpEF, circulating adiponectin levels may be paradoxically elevated, yet higher concentrations are linked to poorer functional capacity and outcomes—a pattern widely interpreted as ineffective downstream signaling in chronic inflammatory states rather than proper protection [2,5]. Thus, low adiponectin in obesity and inflamed EAT likely initiates the HFpEF cascade, whereas later hyperadiponectinemia marks catabolic, advanced-stage remodeling rather than benefit [2,5,32]. Furthermore, while obesity is consistently associated with reduced adiponectin concentrations, higher circulating levels in established congestive heart failure are paradoxically linked to worse clinical outcomes. Several non-mutually exclusive explanations have been proposed, including acquired resistance at the adiponectin receptor level, altered downstream signaling in chronic inflammatory states, and reverse causation driven by hemodynamic stress. However, evidence supporting these mechanisms is limited, as interventional studies directly interrogating adiponectin signaling in HFpEF are lacking [128].

A similar context-dependent duality applies to leptin. Experimental data suggest that intact leptin signaling may exert protective effects on the myocardium, whereas chronic hyperleptinemia in humans is associated with endothelial dysfunction, fibrotic remodeling, and adverse metabolic consequences. The inconsistency of observed associations across human studies highlights the importance of leptin resistance, tissue-specific signaling, and disease stage in shaping its cardiovascular effects [129,130,131].

Beyond biological complexity, methodological limitations contribute substantially to existing evidence gaps. Many studies rely on cross-sectional designs, single-time-point biomarker measurements, and heterogeneous assay platforms, which restrict causal interpretation and reproducibility. Furthermore, translational narratives may be disproportionately influenced by recent therapeutic trials, while earlier mechanistic insights and negative findings are less frequently integrated. Greater emphasis on longitudinal designs, standardized biomarker assessment, and prospectively registered analyses is needed to clarify the temporal dynamics and pathogenic relevance of adipokine signaling in HFpEF.

### 3.5. Translational Limitations: Rodent vs. Human Adipokine Biology

Rodent models provide mechanistic insights yet diverge substantially from human HFpEF biology. Adipokine expression patterns differ markedly: mouse HFpEF shows decreased adiponectin, leptin, and resistin, whereas human adipose tissue exhibits unchanged adiponectin/leptin but marked resistin elevation. Gene expression profiling reveals that mouse HFpEF features elevated UCP1 and TNF-α with decreased collagen; human HFpEF shows collagen elevation only in acute decompensation. These divergences reflect differences in disease chronicity, aging, and comorbidity burden, exceeding 95% prevalence in humans but difficult to replicate in mouse models [132,133,134].

Brown adipose tissue thermogenesis exemplifies species-specific regulatory differences. Rodents predominantly employ β3-adrenergic receptor–mediated UCP1 activation for cold-induced thermogenesis, whereas humans express very low β3-AR and instead rely on β1/β2-AR signaling. Human UCP1 is additionally regulated by ERRα independently of β-adrenergic pathways, a regulatory mechanism unclear in mice [127].

Immune cell infiltration shows striking strain-specificity: C57BL/6 mice display elevated Foxp3+ regulatory T cells in adipose tissue, absent in other mouse strains, macaques, and humans. This suggests strain-specific biases in translating inflammatory conclusions. Sex-dependent mitochondrial function and metabolic flexibility differ markedly in mice yet remain inconsistently replicated in humans [135,136].

Leptin signaling illustrates critical mechanistic divergence. Rodents develop leptin resistance through impaired central transport or receptor deficiency; humans exhibit acquired peripheral leptin resistance via increased caveolin-1–mediated suppression of receptor signaling. This distinction has direct therapeutic implications [137].

Finally, most preclinical HFpEF models fail to meet human diagnostic criteria, with single-stressor interventions producing incomplete phenotypes. Multifactorial models better approximate disease, yet show lower left-ventricular end-diastolic pressure than human HFpEF. These convergent gaps—molecular, physiological, and systemic—underscore that rodent studies remain hypothesis-generating rather than definitively predictive of human HFpEF biology [138].

## 4. Translational Implications: From Biomarkers to Therapeutic Targets

### 4.1. The Adipokine Signature as a Diagnostic and Prognostic Framework

Adipokines are now viewed less as standalone markers and more as part of a broader endocrine system that mirrors how metabolic imbalance, inflammation, and tissue remodeling interact [4,5]. Recognizing this interconnectedness has begun to change their clinical relevance: instead of highlighting single derangements, specific adipokine patterns increasingly help delineate heart-failure phenotypes and reveal patients whose metabolic milieu confers a higher risk of adverse outcomes.

Within this framework, omentin-1 has emerged as a particularly informative marker. In HFpEF, circulating concentrations are consistently reduced, and lower levels correspond with greater systemic inflammation and impaired diastolic function [139]. Notably, in adults aged 70–80 years, omentin-1 outperformed NT-proBNP as a diagnostic discriminator, suggesting that age modifies biomarker performance in a syndrome where comorbidities frequently dilute natriuretic peptide specificity [139]. This finding, however, is derived from a single study population, and although omentin-1 demonstrated incremental discriminatory performance, biomarker claims of superiority require replication in independent populations with standardized assays and proof of added value beyond conventional clinical and echocardiographic parameters.

Adiponectin provides an instructive example of the complexity of adipokine interpretation. In the Heart and Soul Study, individuals in the highest adiponectin quartile had a substantially greater risk of heart failure hospitalization and mortality, despite adiponectin’s well-established metabolic and endothelial benefits [140]. This “adiponectin paradox,” confirmed by meta-analysis, may reflect reverse causation: Mendelian-randomization analyses indicate that increases in NT-proBNP can drive elevations in adiponectin, masking the underlying direction of pathophysiology [141,142].

The integration of adipokines improves risk classification. The OROME score, which combines orosomucoid and omentin-1, indicated a high-risk grouping and enhanced prognostic differentiation when incorporated with NT-proBNP [143]. Multimarker techniques reflect the diverse biology of HFpEF, where metabolic, inflammatory, and haemodynamic signals intersect.

Among emerging biomarkers, GDF15 provides one of the most consistent links between metabolic stress and both cardiac and skeletal-muscle dysfunction. Elevated concentrations are associated with diastolic stiffness, reduced exercise capacity, and increased mortality across the heart-failure spectrum [66,67]. Because GDF15 reflects mitochondrial distress in the myocardium and in skeletal muscle, it represents a molecular hinge between sarcopenia and HFpEF [68].

### 4.2. Leptin Resistance and the Therapeutic Paradox in Sarcopenic Heart Failure

As detailed in Section 3.3, chronic hyperleptinemia in obesity and HFpEF promotes endothelial dysfunction and myocardial fibrotic remodeling, effects that are amplified by acquired leptin resistance. Thus, translational initiatives are progressively emphasizing the restoration of leptin sensitivity instead of merely decreasing leptin levels. Inhibitors of intracellular negative regulators, specifically SOCS3 and PTP1B, demonstrate potential as leptin-sensitizing agents in preclinical models [144,145,146,147,148]. In addition to peripheral pathways, central nervous system circuits may also provide a therapeutic opportunity. Experimental work demonstrates that activation of hypothalamic leptin and melanocortin-4 receptor (MC4R) pathways confers cardioprotection after myocardial infarction, even in the presence of peripheral leptin resistance [149,150]. The availability of the MC4R agonist setmelanotide, approved for rare genetic obesity syndromes, underscores the translational potential of neuroendocrine modulation [151].

### 4.3. GLP-1 Receptor Agonists: Rebalancing the Adipokine Network

The clinical success of GLP-1 receptor agonists (GLP-1RAs) has brought adipokine biology directly into therapeutic practice. Meta-analyses show meaningful reductions in heart-failure hospitalization and cardiovascular mortality, with powerful effects in patients with obesity or diabetes [152].

Two pivotal trials have clarified these benefits in HFpEF. In STEP-HFpEF, once-weekly semaglutide improved symptoms, exercise capacity, body composition, and inflammatory profiles in patients with obesity-related HFpEF [153]. The SUMMIT trial subsequently demonstrated that tirzepatide reduced the composite of cardiovascular death or worsening heart failure while producing substantial gains in health status and physical function [154]. Complementing these findings, the prespecified SELECT analysis confirmed that semaglutide has a favorable impact on heart-failure outcomes in patients with prevalent HF [155].

Mechanistically, GLP-1RAs appear to restore balance within the adipokine axis rather than simply induce weight loss. Imaging studies indicate that GLP-1 receptor agonists selectively diminish visceral and epicardial adipose tissue, which disproportionately contribute to pro-inflammatory adipokine secretion and paracrine coronary microvascular dysfunction [156]. Experimental findings indicate increases in adiponectin and decreases in inflammatory adipokines post-treatment; nevertheless, comprehensive human datasets linking depot regression to adipokine trajectories are scarce [28,32,41,156].

A clinically significant caveat pertains to the loss of lean mass, which may occur with rapid weight loss. This raises concerns regarding functional deterioration in individuals with or at risk for sarcopenia [157,158]. For these individuals, GLP-1RA must be combined with dietary optimization, resistance exercise, and regular body composition evaluations to preserve skeletal muscle integrity [159].

### 4.4. Targeting Specific Adipokine Pathways: Emerging Therapeutic Strategies

In addition to systemic metabolic treatments, certain adipokines are also being investigated as direct therapeutic targets. Omentin-1 boosts endothelial function via AMPK and Akt activation, improves NO-mediated vasodilation, and increases insulin sensitivity, so it mechanistically corresponds with the microvascular phenotype of HFpEF [44]. In preclinical models, adeno-associated virus-mediated overexpression of omentin-1 enhanced left ventricular function and mitigated ischemia–reperfusion injury by activating SIRT3/FOXO3a, demonstrating the feasibility of adipokine replacement therapy [160].

The FGF21 pathway demonstrates comparable translational momentum. FGF21, a hepatokine/adipokine induced by stress, facilitates mitochondrial repair and enhances metabolic flexibility [55,60]. Early human trials of long-acting FGF21 analogs have improved dyslipidemia and hepatic steatosis, and cardiac outcome studies are underway [161].

Finally, adiponectin-receptor agonism offers a means to bypass the adiponectin paradox. Small-molecule AdipoR agonists activate AMPK and PPAR-α independently of circulating adiponectin levels, potentially restoring metabolic–vascular coupling in states of receptor resistance [162]. Preclinical studies show favorable effects on endothelial function, myocardial fibrosis, and exercise capacity, though human translation remains nascent [43,55,56,161,162].

### 4.5. Multi-Modal Biomarker Integration and Precision Medicine Approaches

Because adipokine signaling intersects metabolic, inflammatory, vascular, and musculoskeletal pathways, translation to clinical practice requires multidimensional analysis. Machine-learning platforms that integrate adipokines with echocardiographic parameters, imaging of ectopic fat, and conventional biomarkers show promise for identifying HFpEF subtypes and predicting therapeutic response [163]. Network-based analyses further reveal modular adipokine clusters—metabolic, inflammatory, fibrotic—that may guide individualized treatment strategies.

Point-of-care panels capable of multiplex adipokine measurement are in development [164]. When combined with digital health tools, such platforms could facilitate real-time evaluation of adipokine trajectories and permit earlier identification of biological decompensation.

### 4.6. Challenges and Future Directions in Adipokine-Based Therapeutics

Despite promising molecular and preliminary clinical indications, adipokine-targeted treatments continue to encounter significant translational obstacles. A primary issue stems from the pleiotropic effects of several adipokines: altering a single molecule can have unanticipated systemic consequences. Leptin serves as a salient example; in addition to modulating appetite and energy expenditure, it exerts significant sympathoexcitatory effects that enhance renal and systemic sympathetic output and facilitate platelet activation and endothelial dysfunction, thereby fostering a prothrombotic phenotype [165,166]. These complex effects highlight the necessity for techniques targeting adipokines to focus on specific tissues rather than employing systemic inhibition. Emerging approaches-including nanoparticle-based delivery, receptor-targeted vectors, and aptamer-mediated ligand modulation—are being explored to localize therapeutic effects and minimize off-target toxicity [167,168].

A further translational obstacle pertains to timing. HFpEF advances gradually from metabolic dysfunction to significant haemodynamic impairment. Nevertheless, longitudinal studies monitoring the dynamic changes in adipokine profiles over this continuum are scarce. Expert consensus underscores the need for sequential biomarker acquisition to elucidate disease trajectories, enhance HFpEF features, and identify optimal therapy periods for adipokine modulation efficacy [3,169]. These efforts are especially relevant as adipokines are increasingly acknowledged not only as biomarkers but also as molecular intermediates linking visceral fat, inflammation, and cardiac stiffness [4].

Finally, given the interconnected nature of metabolic and inflammatory pathways in HFpEF, single-target interventions may prove insufficient. Dual-incretin agonists such as tirzepatide exemplify the benefits of coordinated metabolic modulation: activation of both GIP and GLP-1 receptors produces superior improvements in weight, glycemic control, and inflammatory tone compared with GLP-1 receptor agonism alone [170,171]. Preclinical evidence supports biological synergy between GIP and GLP-1 signaling, reinforcing the rationale for combination incretin therapy in cardiometabolic disease [172].

These observations suggest that rational combinations—pairing, for example, AdipoR agonism with omentin-1 restoration or GLP-1RA therapy with targeted anti-inflammatory adipokine modulation—may represent the next step in rebalancing the adipokine axis in HFpEF.

### 4.7. The Adiponectin Paradox: Implications for Therapeutic Development

The adiponectin paradox, wherein elevated concentrations are associated with worse outcomes despite the molecule’s inherent cardioprotective actions, epitomizes the challenge of translating adipokine biology [173]. The paradox is consistent across assays measuring total or high-molecular-weight adiponectin [174]. Meta-analytic data show that each standard deviation increase in adiponectin corresponds to elevated all-cause and cardiovascular mortality [174].

Adjustment for natriuretic peptides attenuates this association, suggesting that adiponectin may, in part, reflect hemodynamic stress rather than protection [175]. Furthermore, the association is contingent upon context: U-shaped in individuals without cardiovascular disease, but linear in cases of established heart failure [176]. These data endorse treatment techniques aimed at restoring receptor sensitivity instead of indiscriminately elevating adiponectin levels.

### 4.8. Implications for Clinical Practice and Guidelines

These mechanistic and translational insights are starting to impact clinical management. Integrating adipokine panels into risk-stratification algorithms may help identify patients who would benefit from enhanced metabolic management, as multimarker techniques combining inflammatory and protective adipokines have shown predictive value beyond that of natriuretic peptides alone [143]. The marked efficacy of GLP-1 receptor agonists in obese HFpEF—now confirmed in randomized trials such as STEP-HFpEF —supports the concept that therapies capable of rebalancing the adipokine axis can be selectively targeted to biomarker-defined phenotypes [177].

Monitoring protocols must progress along with these treatment advancements. An elevation in adiponectin, despite the absence of evident clinical decline, may signify heightened haemodynamic stress and foreshadow adverse outcomes, as observed in extensive community cohorts and heart failure populations [176]. In contrast, the restoration of omentin-1 levels has been linked to improved prognosis and may indicate the reestablishment of microvascular equilibrium [178]. Monitoring the leptin-to-adiponectin ratio—an established indicator of cardiometabolic dysregulation and vascular risk—may enhance the adjustment of metabolic therapy and the identification of developing treatment resistance [179].

Ultimately, given that HFpEF lies at the intersection of fat and muscle atrophy, sarcopenia must be systematically assessed alongside adipokine biology. Integrating adipokine profiling with objective assessments of body composition, such as bioimpedance analysis or DEXA, helps ensure that metabolic therapies reduce pathological adiposity while preserving skeletal-muscle mass and function [180]. An integrated overview of the key adipokines, their molecular mechanisms, and translational relevance is provided in Table 1.

## 5. Conclusions

The expanding characterization of adipokine dysregulation in obesity-associated HFpEF provides a mechanistic framework linking metabolic inflammation to myocardial stiffening and muscle atrophy. However, substantial gaps remain between current scientific understanding and clinical application. Future research must prioritize several critical areas. First, longitudinal biomarker-driven studies measuring adipokine trajectories during disease progression and in response to therapeutic interventions are essential to establish causal pathways and identify treatment-responsive subgroups. Current evidence relies heavily on cross-sectional associations and single time-point measurements; prospective studies employing multi-adipokine panels with standardized assays would clarify temporal dynamics and mechanistic roles. Second, precision medicine approaches utilizing baseline adipokine profiles to predict treatment response represent a promising but unvalidated strategy requiring clinical trial validation. Third, therapeutic development remains in early stages, while GLP-1 receptor agonists show functional improvement in HFpEF, their adipokine-specific mechanisms are poorly characterized, and adipokine-targeted interventions (adiponectin mimetics, leptin antagonists, GDF15 modulators) lack phase 2–3 efficacy data in humans. Combination approaches targeting multiple adipokine pathways simultaneously, rather than single-target inhibition, may prove more effective given adipokine network redundancy. Fourth, tissue-specific delivery technologies enabling selective enhancement of protective adipokines in coronary endothelium while minimizing off-target effects remain underdeveloped. Fifth, mechanistic biomarker endpoints—including circulating adipokine measurements, microvascular function assessments via coronary flow reserve, and advanced cardiac imaging, should be incorporated alongside clinical outcomes in future trials to establish causal links rather than correlations. Finally, real-world pragmatic trials enrolling unselected HFpEF populations (elderly, renal disease, multiple comorbidities) are needed to assess generalizability of controlled trial results.

Collectively, these advances would transform adipokine science from its current descriptive and mechanistic phase into a clinically actionable framework enabling personalized, biologically informed HFpEF management.

## Figures and Tables

**Table 1:** Molecular Characteristics, Pathogenic Roles, and Translational Implications of Key Adipokines and Adipokine-Related Pathways in Obesity- and Sarcopenia-Associated HFpEF.

Adipokine/Pathway	Primary Molecular Actions	Pathogenic Roles in Obesity–Sarcopenia–HFpEF	Translational and Clinical Implications	References
Omentin-1	Activates AMPK and Akt pathways; increases endothelial NO; improves insulin sensitivity; stimulates SIRT3/FOXO3a signaling	Reduced levels contribute to microvascular dysfunction, systemic inflammation, and impaired diastolic relaxation	Diagnostic biomarker (outperforms NT-proBNP in the elderly); AAV-mediated omentin-1 therapy improves LV function and reduces ischemia–reperfusion injury	[30,42,43,139,160]
Adiponectin	Activates AMPK and PPAR-α; enhances fatty acid oxidation; anti-inflammatory; improves NO bioavailability	Elevated levels paradoxically predict higher mortality and HF hospitalization; influenced by NT-proBNP-driven reverse causality	AdipoR agonists bypass circulating adiponectin; potential therapy for restoring metabolic–vascular coupling	[2,11,12,140,141,142,173,174]
GDF15	Stress-induced cytokine; reflects mitochondrial dysfunction; regulates catabolic signaling via GFRAL	Elevated levels associated with diastolic stiffness, exercise intolerance, and increased mortality; a mechanistic bridge between muscle wasting and HFpEF	Strong prognostic biomarker; potential therapeutic target pending tissue-specific modulation strategies	[61,62,63,64,65,66,67,68]
Leptin	Activates JAK/STAT and TGF-β1 pathways; promotes sympathetic activation; suppresses ghrelin; induces SOCS3 feedback inhibition	Hyperleptinemia drives myocardial fibrosis, endothelial dysfunction, and skeletal-muscle catabolism	Leptin-sensitizing strategies (SOCS3/PTP1B inhibition); central MC4R activation provides cardioprotection	[84,85,131,165,166]
Leptin Resistance Mechanisms	SOCS3 inhibits leptin receptor signaling; PTP1B dephosphorylates JAK2; impaired BBB transport reduces central leptin action	Sustained leptin elevation with reduced signaling effectiveness; contributes to catabolic muscle loss and metabolic inflammation	SOCS3/PTP1B inhibitors and MC4R agonists (e.g., setmelanotide) under investigation	[144,145,146,147,148]
GLP-1 Receptor Agonists (GLP-1RA)	Reduce visceral and epicardial fat; increase adiponectin; decrease pro-inflammatory adipokines; improve mitochondrial function	Improve systemic inflammation, microvascular function, and metabolic balance; potential for lean-mass loss in sarcopenic patients	STEP-HFpEF and SUMMIT trials support HFpEF benefit; require monitoring of lean mass and body composition	[152,153,154,155,156,157,158]
FGF21	Enhances mitochondrial repair; increases metabolic flexibility; reduces ER stress; regulates glucose and lipid metabolism	Counteracts metabolic inflexibility and lipotoxicity relevant to HFpEF and sarcopenia	Long-acting analogs show metabolic benefits; cardiac outcome trials ongoing	[55,56,57,58,59,60,161]
AdipoR Agonists	Activate AMPK/PPAR-α independently of adiponectin; promote mitochondrial biogenesis; improve endothelial NO	Overcome adiponectin resistance; reduce myocardial fibrosis; improve exercise capacity in preclinical HF models	Potential therapeutic class for metabolic–vascular restoration	[11,128,162,173]
Visceral and Epicardial Adipose Tissue Pathways	Secrete IL-6, TNF-α, resistin; suppress protective adipokines; induce microvascular inflammation	Promote coronary microvascular dysfunction and stiffening, central to HFpEF phenotype	GLP-1RAs selectively reduce these depots; possible imaging–biomarker integration	[22,24,41,82,83]
SOCS3/PTP1B Signaling	Negative regulators of leptin receptor JAK/STAT signaling	Induce leptin resistance and perpetuate hyperleptinemic inflammation	Pharmacologic inhibition restores leptin sensitivity in early research	[144,145,146,147]
Melanocortin-4 Receptor (MC4R)	Central regulator of energy balance and sympathetic tone; interacts with leptin–POMC axis	Activation confers cardioprotection despite peripheral leptin resistance	Setmelanotide demonstrates clinical feasibility of MC4R targeting	[129,149,151]
Inflammatory Adipokines (resistin, TNF-α, IL-6)	Activate NF-κB, JAK/STAT, MAPK pathways; promote oxidative stress	Contribute to myocardial fibrosis, endothelial dysfunction, and muscle catabolism	Modulated indirectly through weight-loss therapies and GLP-1RAs	[25,26,32,38]
Multimarker Scores (e.g., OROME)	Combine inflammatory and protective adipokines	Improve HFpEF risk stratification beyond natriuretic peptides	Incorporation into precision-medicine algorithms	[143]
Ectopic Fat + Adipokine Integration	Links regional fat depots to unique adipokine signatures	Explains phenotypic diversity in HFpEF	Supports machine-learning and multimodal biomarker platforms	[41,82,83,120,163]

## Data Availability

No new data were created or analyzed in this study. Data sharing is not applicable to this article.
